# Supplementary material for: Systemic Oxidative Stress in Severe Early-Onset Fetal Growth Restriction Associates with Concomitant Pre-Eclampsia, Not with Severity of Fetal Growth Restriction
Source: Antioxidants (Basel). 2023 Dec 26;13(1):46. doi: 10.3390/antiox13010046 (PMC10812523; doi:10.3390/antiox13010046)
Supplement: Supplementary file 1 [file antioxidants-13-00046-s001.zip › antioxidants-2758527-supplementary.pdf]

antioxidants

MDPI

Supplementary Materials

Table S1. Correlations between plasma free thiols at study inclusion. Correlations between continuous variables were calculated using Spearman's rank correlation coefficients (ϱ).

	ϱ	*p* value
Age (years)	−0.064	0.521
BMI (mean)	−0.145	0.142
Maternal smoking (n)	0.132	0.383
Gestational age at randomization (median)	−0.343	<0.001
Ultra-sonographic examination results<br>· EFW (gram)<br>· FAC (mm)	−0.450<br>−0.404	<0.001<br><0.001
Fetal sex	−0.075	0.473
Notching uterine artery	0.010	0.930
Pulsatility index<br>· Umbilical artery<br>· Middle cerebral artery	 −0.047<br>−0.101	 0.648<br>0.325
End-diastolic flow<br>· Positive<br>· Absent	−0.073<br>0.076	0.574<br>0.674
Low dose aspirin use before 16 weeks	−0.058	0.578
Pregnancy induced hypertension at inclusion	−0.085	0.383
Pregnancy induced hypertension at delivery	−0.281	0.004
Pre-eclampsia at inclusion	−0.505	<0.001
Pre-eclampsia at delivery	−0.313	0.002
Blood pressure<br>· Systolic mmHg<br>· Diastolic mmHg	−0.348<br>−0.266	0.001<br>0.012
Proteinuria	−0.660	0.004
Onset of labor<br>· Spontaneous<br>· Induction of labour	−0.085	0.384

· Prelabour caesarean section<br>· Termination of pregnancy		
Indication for induced delivery<br>· Maternal indication<br>· Fetal indication	0.270	0.007
Mode of delivery<br>· Vaginal<br>· Caesarean section	0.055	0.571
Stillbirth	−0.051	0.603
Cord pH arterial	0.011	0.953
Cord pH venous	−0.123	0.495
Birthweight (grams)	0.056	0.563
Birthweight ratio	−0.149	0.131
Length (cm)	−0.103	0.473
Head circumference (cm)	0.159	0.310
Placental weight (grams)	0.090	0.394
Persistent pulmonary hypertension (PPHN)	−0.002	0.989
Necrotizing enterocolitis (NEC)	0.027	0.818

Table S2. Clinical study cohort characteristics and the original cohort. Clinical characteristics of the current cohort and the original cohort of the Dutch STRIDER study. Data are presented in proportions n with corresponding percentages (%) or Standard Deviation (), unless stated otherwise in the table. Abbreviations: BMI, body mass index (calculated as weight in kilograms divided by height in meters squared). Estimated Fetal Weight (EFW) was calculated by using the Hadlock equation. Preeclampsia was defined as gestational hypertension accompanied by one or more of the following new-onset conditions at or after 20 weeks' gestation: 1. Proteinuria 2. Other maternal organ dysfunction 3. Uteroplacental dysfunction (32) # Study medication was given after collection of maternal blood.

**Baseline at inclusion**	**This study (n = 108)**	**Original cohort (11) (n = 216)**
Maternal		
Age (years)	32 (±5)	31 (±5)
BMI (kg/m²)	24.5 (±6.1)	25 (±5.8)
Ethnicity<br>- Caucasian<br>- African<br>- Asian	 80 (79.2%)<br>11 (10,9%)<br>3 (3.0%)	 170 (78.7%)<br>18 (8.3%)<br>7 (3.2%)

- Other	14 (13%)	19 (8.7%)
Current smoker	8 (7.6%)	18 (8.3%)
Low dose aspirin usage	10 (9.5%)	23 (10.6%)
Gestational hypertension	23 (21.9%)	46 (21.2%)
Pre-eclampsia	21 (20%)	49 (22.6%)
Systolic blood pressure (mm Hg)	134 (±41)	132 (±20)
Diastolic bloodpressure (mm Hg)	84 (±24)	83 (±15)
Sildenafil #	52 (50.5%)	108 (50%)
Fetal		
Gestational age (weeks + days)	24 + 3 (23 + 3 to 25 + 3)	24 + 4 (23 + 4 to 25 + 4)
Estimated fetal weight (grams)	466 (±192.3)	461 (±173)
Abdominal circumference (mm)	163.7 (±26.7)	165 (±26)
Umbilical artery *p* >p95	61 (59.2%)	104 (48,1%)
Middle cerebral artery pulsatility index <p5	58 (59.8%)	90 (41.6%)
Birth outcomes		
Gestational hypertension	73 (67.6%)	146 (67.9%)
Gestational hypertension developed after inclusion	40 (37.0%)	N/A
Pre-eclampsia	43 (39.8%)	86 (40%)
Pre-eclampsia developed after inclusion	22 (20.4%)	N/A
Onset of labor		
Spontaneous	9 (8.6%, 5 stillbirths)	18 (8.3%)
Induction of labor	35 (32.4%, 24 stillbirths)	58 (27.6%)
Prelabour cesarean section	61 (58.1%, 0 stillbirths)	166 (77.2%)
Mode of delivery		
Spontaneous vaginal delivery	40 (38.1%, 29 stillbirths)	N/A
Instrumental vaginal delivery	1 (1.0%, 0 stillbirths)	N/A

Caesarean section	64<br>(61%, 0 stillbirths)	N/A
Neonatal outcomes		
Gestational age at delivery (weeks + days)	28 + 3 (27 + 5 to 29 + 1)	29 + 3 (28 + 5 to 30 + 1)
Birthweight (g)	811 (±510.5)	856.5 (±582)
Birthweight ratio	0.5583 (±0.136)	N/A
Stillbirth	29 (27.6%)	52 (24.2%)
Male sex	58 (53.7%)	110 (51.2%)
Cord pH arterial	7.3 (±0.10)	N/A
Cord pH venous	7.4 (±0.13)	N/A
Birthweight below p10	86 (96.6%)	142 (65.6%)
Birthweight below p3	77 (86.5%)	57 (27%)
Persistent pulmonary hypertension	8 / 79 (10.1%)	N/A
Necrotizing enterocolitis	9 / 79 (11.3%)	15 / 163 (9.3%)
Early-onset sepsis	5 / 79 (6.3%)	N/A
Late-onset sepsis	20 / 79 (25.3%)	N/A
Periventricular leukomalacia grade ≥ 3	0 / 79 (0%)	0 /163 (0%)
Intraventricular hemorrhage grade ≥ 3	2 / 79 (2.5%)	5 /163 (3.1%)
Neonatal death	18 / 79 (22.7%)	32 / 163 (19.1%)
